# Chimeric JAK2 Kinases Trigger Non-uniform Changes of Cellular Metabolism in BCR-ABL1-like Childhood ALL

**DOI:** 10.1097/HS9.0000000000000946

**Published:** 2023-08-23

**Authors:** Julius Lukes, Eliska Potuckova, Natividad Alquezar-Artieda, Ivana Hermanova, Sladjana Kosanovic, Katerina Hlozkova, Meritxell Alberich Jorda, Jan Zuna, Jan Trka, Daniel A. Tennant, Martin Stanulla, Marketa Zaliova, Julia Starkova

**Affiliations:** 1 CLIP - Childhood Leukaemia Investigation Prague, Czech Republic; 2 Department of Paediatric Haematology and Oncology, Second Faculty of Medicine, Charles University, Prague, Czech Republic; 3 Laboratory of Hemato-oncology, Institute of Molecular Genetics of the Czech Academy of Sciences, Prague, Czech Republic; 4 University Hospital Motol, Prague, Czech Republic; 5 Institute of Metabolism and Systems Research, College of Medical and Dental Sciences, University of Birmingham, United Kingdom; 6 Pediatric Hematology and Oncology, Hannover Medical School, Hannover, Germany

Almost a century ago, Otto Warburg described increased glucose uptake and induced glycolysis in malignant cells, a metabolic alteration, which is now considered a general feature of tumor cells.^1^ Aerobic glycolysis (so-called Warburg effect) was regarded as a privileged energetic pathway despite its lower energy production in comparison to oxidative phosphorylation (OXPHOS), the bioenergetic process generally used in normal cells. However, recent studies suggest that malignancies are metabolically very heterogeneous, with some relying on glycolysis while other preferentially on OXPHOS.^2,3^

Malignant transformation is a process characterized by the acquisition of multiple genetic aberrations that enable uncontrolled proliferation of cancer cells and their invasion of other tissues. Some variants affecting cellular signaling may be essential in altering cellular metabolism in favor of increased energetic demands of malignant cells.^4–6^ The investigation of different metabolic changes driven by specific genetic backgrounds of individual cancers can contribute to our better understanding of the process of tumorigenesis and identify novel cancer vulnerabilities possibly exploitable in therapy.

In this study, we focused on acute lymphoblastic leukemia (ALL) and studied the impact of specific genetic aberrations on the metabolism of leukemic cells. ALL represents the most common pediatric cancer with an overall good but strikingly heterogeneous prognosis. One of the subgroups with less favorable prognosis is defined as *BCR-ABL1*-like (*Ph*-like) ALL and accounts for up to 10%–15% of all pediatric ALL cases and for up to 20%–25% of ALLs in adults.^7,8^ Unlike the *BCR-ABL1*-positive ALLs, the *BCR-ABL1*-like ALLs do not contain the *BCR-ABL1* fusion, yet their gene expression profile is similar to that of *BCR-ABL1*-positive ALL. The majority of *BCR-ABL1*-like cases harbor primary genetic lesions affecting kinase signaling, one of the examples being *JAK2* fusions causing constitutive activation of the Janus kinase 2 (JAK2). The most common fusion partner of *JAK2* in *BCR-ABL1*-like ALL is *PAX5*; however, the repertoire of fusion partners is still growing as less frequent fusions continue to be discovered.^8^ In line with this fact, we recently identified a novel *JAK2* fusion, *NPAT-JAK2*, in a patient with *BCR-ABL1*-like ALL.^9^ Here we describe this fusion, study its impact on JAK/STAT kinase signaling and its oncogenic potential, and compare it with that of *PAX5-JAK2*. Importantly, we describe the impact of both fusions on cellular metabolism.

Molecular characterization of the *NPAT-JAK2* fusion gene showed that, similarly to other described *JAK2* fusions, *NPAT-JAK2* involves an intact JAK2 kinase domain (for details of *NPAT-JAK2* sequence and structure see Suppl. Figure S1). In transiently transfected HEK293T cells, *NPAT-JAK2* was translated into an in-silico-predicted chimeric protein (molecular weight 74 kDa), which was localized both in the cytoplasm and the nucleus (predominantly in the cytoplasm) and was phosphorylated on tyrosines corresponding to Y1007/Y1008 of wild-type JAK2 (Figure 1A). Introduction of an inactivating mutation (*NPAT-JAK2^K882E^*) to the ATP-binding site within the *JAK2* moiety (corresponding to K882 of wild-type *JAK2*) prevented NPAT-JAK2 phosphorylation, which strongly suggested that the chimeric protein was autophosphorylated upon constitutive activation of the JAK2 kinase domain. Thus, we confirmed that the JAK2 moiety within NPAT-JAK2 preserved its ATP-binding and catalytic function (Figure 1A).

**Figure 1.**
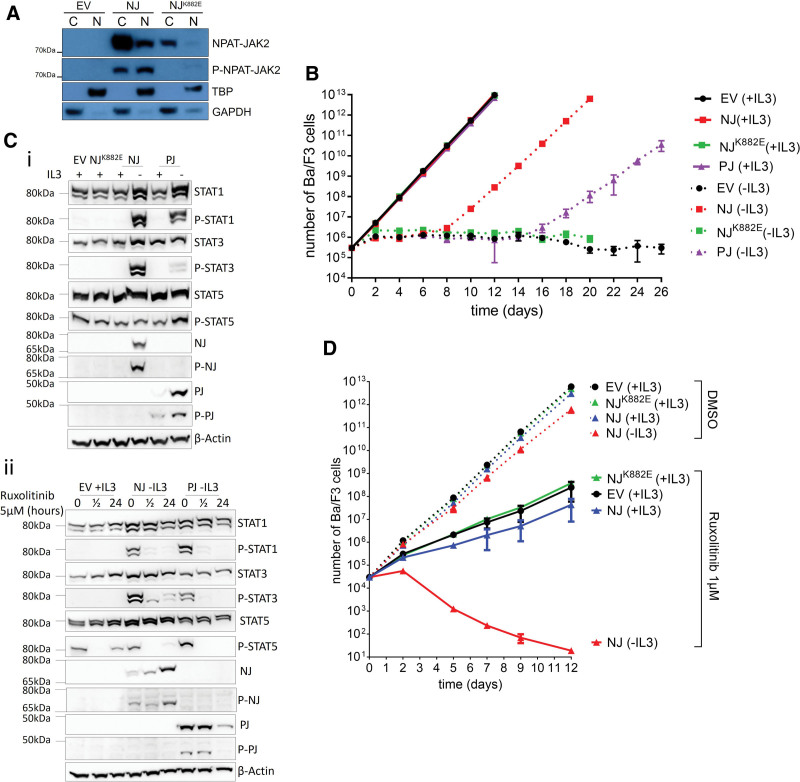
**Functional characterization of NPAT-JAK2 and energetic changes induced by NPAT-JAK2 and PAX5-JAK2 in Ba/F3 cells.** (A) In-silico predicted NJ chimeric protein was expressed and phosphorylated in transfected HEK293T. It was localized both in the nucleus (N) and the cytoplasm (C). Mutational inactivation of an ATP-binding site (NJ^K882E^) and the EV served as controls. GAPDH and TBP worked as loading controls. (B) The proliferation curve was assessed by counting viable Ba/F3 cells transduced with EV, NJ, NJ^K882E^, or PJ, grown with IL-3 or independently on IL-3. The proliferation of sorted Ba/F3 cells was measured every other day. Trypan blue was used to exclude dead cells. Values represent the mean out of 3 biological replicates. (C) (i) Detection of NJ and PJ, phosphorylated forms p-NJ and p-PJ and downstream effectors STAT1, STAT3, and STAT5 by western blot in Ba/F3 cells with or without IL-3 supplementation. B-actin was used as a loading control. (ii) Effect of ruxolitinib on protein expression of NJ, PJ, phosphorylated forms p-NJ and p-PJ, and downstream effectors, STAT1, STAT3, and STAT5 by western blot in Ba/F3 cells without IL-3 supplementation. B-actin was used as a loading control. Ruxolitinib, JAK1/JAK2 inhibitor, was used to monitor the activity of JAK2 signaling pathway and its downstream effectors. (D) The effect of JAK1/JAK2 inhibition by ruxolitinib (1 µM) treatment on the proliferation of transduced Ba/F3 cells. The proliferation curve was assessed by counting viable Ba/F3 cells transduced with EV, NJ, NJ^K882E^, grown in the presence (+IL3) or absence (−IL3). Transduced Ba/F3 cells were counted every other day. Trypan blue was used to exclude dead cells. Values represent the mean out of 3 biological replicates. EV = empty vector; GAPDH = glyceraldehyde 3-phosphate dehydrogenase; IL-3 = interleukin 3; NJ = NPAT-JAK2; PJ = PAX5-JAK2; TBP = TATA-binding protein.

We also studied the oncogenic potential of NPAT-JAK2 and its impact on JAK/STAT pathway signaling. Using a lentiviral vector, *NPAT-JAK2* was introduced into IL-3-dependent Ba/F3 cells, where it exhibited prosurvival and proproliferative capacities upon IL-3-withdrawal (Figure 1B). This effect was not reproduced in the *NPAT-JAK2*^K882E^ mutant cells, indicating its dependence on the NPAT-JAK2 kinase activity. Transformation of Ba/F3 cells by *NPAT-JAK2* was significantly faster compared with *PAX5-JAK2* (Figure 1B). Interestingly, the transformed *PAX5-JAK2* cells under IL-3 starvation (Figure 2Dii) showed a slower growth rate than cells cultured in the presence of IL-3 while such a difference was not observed in the *NPAT-JAK2* model (Figure 2Di).^10^ These results suggest a stronger oncogenic potential of *NPAT-JAK2*.

**Figure 2.**
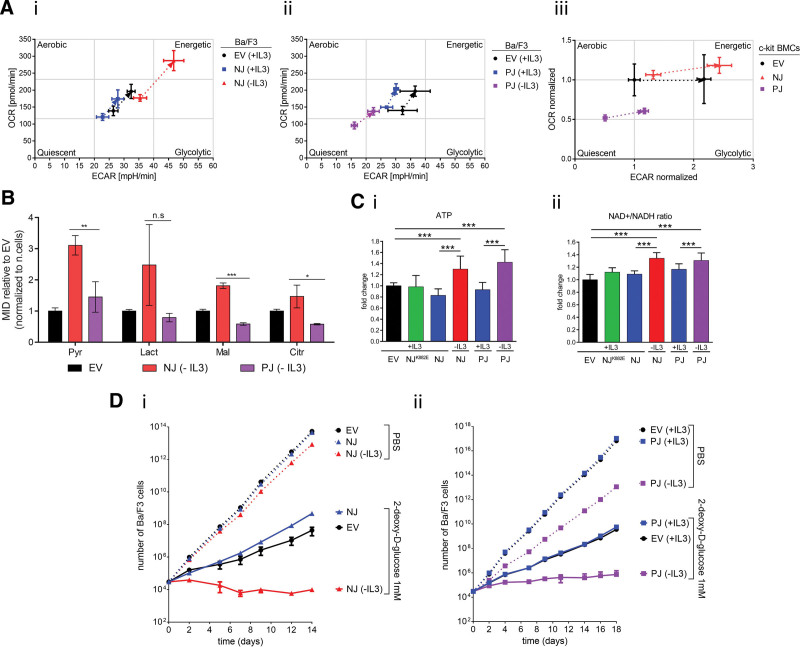
**Metabolic and energetic changes induced by NPAT-JAK2 and PAX5-JAK2 in Ba/F3 cells.** (A) Cell energy phenotype profile was determined in NPAT-JAK2 (i) and PAX5-JAK2 (ii) in Ba/F3 cells, and NPAT-JAK2 and PAX5-JAK2 (iii) in c-kit BMCs. The dotted line connects measurements between basal steady-state conditions and stress induction by oligomycin and FCCP. To avoid variability of the results from c-kit BMCs caused by multiple independent BMCs isolation and instability of c-kit cells in *in vitro* culture, we normalized all measured values to basal levels of OCR/ECAR of c-kit EV BMCs. Arrow represents the direction from basal steady- to stress-state induction. Graph shows the representative replicate. All measurements were performed in biological triplicate. (B) MID of intracellular metabolites detected in transformed Ba/F3 cells cultured for 24 h with labeled glucose, 13C-glucose. Results were normalized to number of cells and represented as relative to EV. Measurement was performed in biological triplicate. Unpaired *t* test with Welch´s correction was applied to compare transduced Ba/F3 cells (*P < 0.05, **P < 0.01, ***P < 0.001, n.s: not significant). (C) ATP production (i) and NAD+/NADH ratio (ii) are significantly higher in NPAT-JAK2- and PAX5-JAK2- transformed Ba/F3 cells. Graph represents fold change to the values of EV. Mann-Whitney *U* test (***P < 0.001). Values represent the mean out of 4 biological replicates, each performed in a technical triplicate, for ATP measurement, and 3 biological replicates, each performed in a technical octuplicate, for the NAD+/NADH ratio. (D) (i, ii) The effect of 2-deoxy-D-glucose (1 mM) treatment on proliferation of transduced Ba/F3 cells. The proliferation curve was assessed by counting viable Ba/F3 cells transduced with EV, NJ, and PJ, grown in the presence (+IL3) or absence (−IL3). Sorted Ba/F3 cells were counted every other day. Trypan blue was used to exclude dead cells. All values represent the mean out of 3 biological replicates. c-kit BMCs = c-kit-positive bone marrow cells; Citr = citrate; ECAR = extracellular acidification rate; FCCP = carbonyl cyanide-p-trifluoromethoxyphenylhydrazone; IL-3 = interleukin 3; Lact = lactate; Mal = malate; MID = mass isotopomere distribution; OCR = oxygen consumption rate; Pyr = pyruvate.

Western blot analysis of the *NPAT-JAK2*-transformed Ba/F3 cells showed increased phosphorylation of STAT1, STAT3, and STAT5 (Figure 1C; Suppl. Figure S2), which was inhibited by the JAK1/2 inhibitor Ruxolitinib (Figure 1Cii; Suppl. Figure S3). Importantly, Ruxolitinib blocked the proproliferative effect of *NPAT-JAK2*-transformed Ba/F3 cells (Figure 1D). Ectopic expression of *PAX5-JAK2* resulted in constitutive phosphorylation of STAT1 and STAT5 and with significantly less phosphorylation of STAT3 comparing to cells with *NPAT-JAK2* expression (Figure 1Ci; Suppl. Figure S3). Analogously, all phosphorylated STATs were decreased after the treatment with JAK2-inhibitors (Figure 1Cii; Suppl. Figure S3), which also inhibited the proliferation of *PAX5-JAK2*-transformed cells.^10^

To determine the metabolic and energetic changes induced by NPAT-JAK2 and PAX5-JAK2, we employed the extracellular flux analysis, which allows real time quantification of extracellular acidification rate (ECAR) representing glycolytic function and oxygen consumption rate (OCR) representing cellular respiration (OXPHOS) in live cells. Using the Cell Energy Phenotype Test, we showed the *NPAT-JAK2*-transformed Ba/F3 cells without IL-3 in comparison to controls with IL-3 supplementation (Ba/F3 cells transduced with empty vector or *NPAT-JAK2*) increase both glycolysis and mitochondrial respiration and acquire a more “energetic” phenotype (Figure 2Ai). This metabolic phenotype shift of *NPAT-JAK2*-transformed Ba/F3 cells was enhanced after stress induction with combination of oligomycin and carbonyl cyanide-p-trifluoromethoxyphenylhydrazone (FCCP) (inhibitor of ATP-synthase and mitochondrial uncoupler, respectively), which enable to determine the cells’ maximal bioenergetic capacity. These findings were accompanied by significantly increased ATP production together with increased NAD+/NADH ratio as assessed by a luminescence assay in the *NPAT-JAK2*-transformed cells when compared with controls (Figure 2C). Intriguingly, *PAX5-JAK2*-transformed cells displayed an opposite metabolic shift toward a more quiescent phenotype with decrease of both OCR and ECAR (Figure 2Aii). Importantly, we employed stable isotope tracing with C_13_-glucose. We confirmed higher activity of glycolysis in NPAT-JAK2-tranduced cells in comparison to PAX5-JAK2 transduced cells. It was represented by increased labeled pyruvate and lactate. Further, we showed that *NPAT-JAK2*-transformed cells increased labeled citrate and malate, intermediates of Krebs cycle (Figure 2B). Nevertheless, their ATP production and NAD+/NADH ratio was similar to that of *NPAT-JAK2*-transformed cells and significantly higher compared with controls (Figure 2C). Furthermore, we used 2-deoxy-D-glucose (2DG), a competitor of glucose (which is one of the main substrates used in bioenergetic pathways of cancer cells) to test the dependence of the *NPAT-JAK2* and the *PAX5-JAK2* transforming potential on bioenergetic rewiring. The proliferation of *NPAT-JAK2*-transformed cells treated with 2DG was dramatically inhibited, compared with control cells. In contrast, the proliferation of *PAX5-JAK2*-transformed cells was affected only moderately (Figure 2D). To support our findings, we introduced *NPAT-JAK2* and *PAX5-JAK2* fusion genes into primary murine c-kit-positive bone marrow cells (c-kit BMCs) and determined their metabolic phenotype. We observed that *NPAT-JAK2* transduced c-kit BMCs had more energetic metabolic phenotype in comparison to more quiescent phenotype detected in *PAX5-JAK2* transduced c-kit BMCs; the result was concordant with the data of transformed Ba/F3 cells (Figure 2Aiii).

Metabolic rewiring, a cancer hallmark, is one of the key mechanisms that underlie tumorigenesis, tumor progression, and chemoresistance.^11^ Although hematological malignancies mainly use aerobic glycolysis, some favor OXPHOS to satisfy their energy demands.^2,12^ Here, we show that lymphoid cells transformed by *NPAT-JAK2*, a novel fusion, which we found in *BCR-ABL1*-like ALL, increase both their glycolytic activity and mitochondrial respiration. These results are in concordance with the previously published study showing elevated glycolysis and increased OXPHOS in an *in vivo* model of myeloproliferative neoplasms driven by *JAK2*-activating mutation.^13^ Surprisingly, we observed that *PAX5-JAK2*-transformed cells change their metabolism in an opposite manner, that is, they lower their glycolytic and respiratory functions. This quiescent metabolic phenotype may potentially mirror the relatively weaker transforming capacity of *PAX5-JAK2* resulting in a lower proliferation rate compared with *NPAT-JAK2*. Suppressed STAT3 signaling in *PAX5-JAK2*-transformed cells could also contribute to lower glycolytic function.^14^ Hypothetically, the metabolic phenotype may be significantly influenced by the biological impact of the *PAX5* moiety. It has been shown that the PAX5-JAK2 protein is localized exclusively in the nucleus and binds to wild-type PAX5 target loci and may activate PAX5 target genes’ expression, although to a lesser extent compared with wild-type PAX5.^10^ Wild-type PAX5 represses glucose and energy metabolism^15^; a partial preservation of this metabolic impact of PAX5 within the PAX5-JAK2 fusion could explain the low glycolytic activity of *PAX5-JAK2*-transformed cells.

Interestingly, despite the differences in glycolytic and OXPHOS activity, both *JAK2* fusions showed increased levels of ATP and NAD^+^/NADH. The elevated ATP production could represent a more general feature of the overall metabolic impact of activating JAK2 lesions, as it was previously also observed in *JAK2*-mutated myeloproliferative neoplasms. The increased ratio of NAD^+^/NADH in *NPAT-JAK2*-transformed cells is likely a result of enhanced mitochondrial respiration, while its nature in the *PAX5-JAK2*-transformed cells is unclear.

In conclusion, we studied the impact of *JAK2* fusions on metabolic rewiring of leukemic cells; we chose two representatives, the most frequent fusion and a novel previously undescribed fusion, and we showed that JAK2 fusions indeed alter energy metabolism of leukemic cells, but surprisingly in a very distinct manner. This discordance between both fusions may result from differences in their overall biological impacts, which are indicated by the variations in transformation potential and subcellular localization and are likely caused by the *JAK2* partner gene character. The *BCR-ABL1*-like ALL includes leukemias with functionally distinct classes of genetic aberrations (such as *ABL* versus *JAK/STAT* class) resulting in a nonnegligible biological but also clinical heterogeneity of this ALL subtype. Yet, our data show that even fusions involving the same kinase gene may exert considerable biological variety, and thus, although we believe that metabolism-targeting drugs may be therapeutically relevant in this unfavorable ALL subtype and deserve further studies, the use of such drugs may require precise tailoring with respect to the specific metabolic impact of individual genetic aberrations.

## AUTHOR CONTRIBUTIONS

JL prepared cell models, performed in vitro cell growth measurements and metabolic characterization experiments, and wrote the article. EP and NAA prepared expression vectors, prepared cell models, performed cell growth measurements, and western blots. SK and MAJ prepared primary murine cells and performed transduction. IH was responsible for sorting and validation of cell models. KH optimized and performed metabolic characterization and analyzed the data. MZ and MS identified and characterized the new fusion. DAT measured GS-MS and helped with interpretation of data. JZ, JT, MS, and MZ participated on study design and data interpretation. JS coordinated the study, designed the experiments, and analyzed the data. JL, NAA, and JS wrote the article. All authors reviewed and approved the article.

## DISCLOSURES

The authors have no conflicts of interest to disclose.

## SOURCES OF FUNDING

The study was supported by a grant from the Ministry of Health (NU22-07-00087), by the Czech Scientific Foundation (GA20-27132S) by the project (Ministry of Health, Czech Republic) for conceptual development of research organization 00064203 (University Hospital Motol, Prague, Czech Republic); the project National Institute for Cancer Research (Programme EXCELES, ID Project No. LX22NPO5102)-Funded by the European Union-Next Generation EU and by the Ministry of Health.

## Supplementary Material


