# Supplementary material for: Chimeric JAK2 Kinases Trigger Non-uniform Changes of Cellular Metabolism in BCR-ABL1-like Childhood ALL
Source: Hemasphere. 2023 Aug 23;7(9):e946. doi: 10.1097/HS9.0000000000000946 (PMC10448929; doi:10.1097/HS9.0000000000000946)
Supplement: Supplementary file 1 [file hs9-7-e946-s001.docx]

**Supplementary Methods:**

**NPAT-JAK2 and PAX5-JAK2 cloning**

The *NPAT-JAK2* full coding sequence was amplified and cloned from the diagnostic bone marrow sample of a patient with *BCR/ABL1*-like ALL diagnosed and treated according to the AIEOP-BFM ALL 2000 treatment protocol in Germany. Informed consent for the research use of biological material was obtained in accordance with the Declaration of Helsinki.

Total RNA from primary diagnostic bone marrow was transcribed into cDNA employing Cloned AMV First-Strand cDNA Synthesis Kit (Thermo Fisher Scientific, Waltham, Massachusetts, USA) with Oligo (dT)20 as a primer. Full-length coding sequence of *NPAT-JAK2* was amplified by PCR using PCR Extender System (5 PRIME GmbH, Germany) and cloned into the pWCC19 vector (kindly provided by Dr. Alberich Jorda, Institute of Molecular Genetics of the ASCR, Czech Republic) using In-Fusion® HD Cloning Kit (Clontech Laboratories, Mountain View, California, USA). The mutation resulting in a substitution of lysine by glutamic acid at the position corresponding to lysine 882 in wild type JAK2 was introduced into *NPAT/JAK2* construct using the QuickChange Site-Directed Mutagenesis Kit (Agilent Technologies, Inc. Santa Clara, California, USA) and the primers listed below. Full length coding sequences of successfully cloned *NPAT/JAK2* and *NPAT/JAK2^K882E^* were analyzed by Sanger sequencing.

| **PCR primers used for cloning and mutagenesis of *NPAT-JAK2*** | | |
| --- | --- | --- |
|  | | |
| ***Primers used for amplification of full length NPAT-JAK2 from cDNA*** | | |
| primer | primer sequence (5' to 3') | |
| forward | CTGCTGTGGTTTTGATCATGTTGT | |
| reverse | GTCATTTCTTTCATCCAGCCATG | |
|  | | |
| ***Primers used for mutagenesis of NPAT-JAK2*** | | |
| primer | | primer sequence (5' to 3') |
| forward | GAGGTGGTCGCTGTCGAAAAGCTTCAGCATAGTACTGAAGAG | |
| reverse | CTCTTCAGTACTATGCTGAAGCTTTTCGACAGCGACCACCTC | |

*PAX-JAK2* full coding sequence was cloned from the plasmid pITR-TCE-Ins-UTR, kindly provided by Dr. Sabine Strehl from Children´s Cancer Research Institute, St. Anna Kinderkrebsforshchung, Vienna. *PAX5-JAK2* was cloned into the pWCC19 vector and the same procedure was followed as in the case of *NPAT-JAK2* fusion gene.

**Cell line cultivation**

The HEK293T, human embryonic kidney cell line, and the NIH-3T3 murine fibroblast cell line (kindly provided by Dr. Alberich-Jorda) were maintained in Dulbecco´s modified Eagles Medium (Thermo Fisher Scientific) supplemented with 10% of heat-inactivated Fetal Bovine Serum (FBS; Biosera, France) and Antibiotic-Antimycotic (Thermo Fisher Scientific). The Ba/F3, interleukin 3 (IL3) dependent murine pro B cell line and leukemic cell line REH (purchased from German Collection of Microorganims and Cell Cultures; DSMZ-Deutsche Sammlung von Mikroorganismen und Zellkulturen GmbH, Germany) were maintained in Roswell Park Memorial Institute medium (Thermo Fisher Scientific) supplemented with 10% of heat-inactivated Fetal Bovine Serum, Antibiotic-Antimycotic and 10 ng/ml IL3 (Sigma-Aldrich, St. Louis, Missouri, USA).

**Transient transfection of HEK293T cells**

The HEK293T cells were seeded on a 6 well plate (600,000 cells per well) 24 hours before the transfection using Lipofectamine2000 reagent (Thermo Fisher Scientific) according to manufacturer’s instructions: 6,4 μg plasmid DNA with 8 μl of Lipofectamine2000 in 2 ml of serum-free medium per well. The transfection efficiency was analyzed by flow cytometry as a percentage of GFP-positive cells. Forty-eight hours after transfection cells were harvested and used for protein analysis.

**Ba/F3 cells transduction**

The lentiviral particles were produced by HEK293T cells transfected with pWCC19 (with or without an insert) together with packaging plasmids of second generation (p-gag-pol, p-VSV-G; kindly provided by Dr. Alberich-Jorda) using Xfect™ Transfection Reagent (Clontech Laboratories). Supernatants containing viral particles were collected 48 and 72 hours after transfection and concentrated on Centricon® Plus-70 Centrifugal Filter Devices (Merck Millipore, Germany) or Lenti-X concentrator (Takara Bio, Shiga, Japan). The titer of the produced virus (multiplicity of infection value – MOI) was assessed in the NIH3T3 cell line using flow cytometric detection of the GFP fluorescence. Transduced Ba/F3 and REH cells were sorted by fluorescence assisted cell-sorter according to their GFP-positivity. Cell sorter was set up to provide a cell fraction purity of more than 98% GFP-positive sorted cells to achieve equivalent transduction efficiency for all models. Moreover, percentage of GFP positive cells was tested regularly before each culture experiment.

**Isolation of murine bone marrow c-kit+ cells**

C57BL/6N WT mice were sacrificed by cervical dislocation, femurs and tibias were isolated, and crunched using pestle and mortar. After obtaining single-cell suspensions, red blood cells were lysed, and BM cells were labeled using a c-kit biotinylated antibody (clone 2B8, Biolegend). Cells were further labeled with anti-biotin magnetic beads (Miltenyi Biotec, Bergisch Gladbach, Germany) and fractionated based on c-kit expression on a MACS separator (Miltenyi Biotec) according to the manufacturer’s protocol.

**Lentiviral transduction of c-kit+ cells**

BM c-kit+ cells were cultured in StemSpan^TM^ SFEM medium (Stemcell Technologies, Vancouver, Canada) supplemented with 10 ng/ml mIL-3, 20 ng/ml hIL-6, 100 ng/ml mSCF, 50 ng/ml mTPO, 100 ng/ml mFlt-3 ligand, antibiotics, and 2% FBS. All cytokines were from Peprotech (London, UK). Lentiviral particles were added to the cultures (20 MOI) for 4 h, washed twice with PBS and cultured for another 48 h before sorting based on GFP expression. Cell sorter was set up to provide a cell fraction purity of more than 98% GFP-positive sorted cells. Moreover, percentage of GFP positive cells was tested regularly before each culture experiment. Sorted cells were expanded and subsequently used in experiments.

**IL3 withdrawal experiments and drug treatment of Ba/F3 cells**

For the IL3 withdrawal experiments, lentivirally-transduced Ba/F3 cells were washed 3 times by PBS and then 300,000 cells were seeded to each well of a 6 well plate (in triplicate) in the media with or without IL3. Cells were counted every second day using trypan blue exclusion to distinguish viable from dead cells. For experiments with ruxolitinib, cells were seeded as described above and treated with 1 µM or 5 μM ruxolitinib (INCB018424; Seleckchem Europe, Germany). During the growth rate analysis, cells were counted and the culture medium containing ruxolitinib was changed every second day. For the western blot analysis, 2.5x10^6^ Ba/F3 cells exposed to 5 μM ruxolitinib for 0.5 h and 24 h were treated by 100 μM sodium orthovanadate (Merck, Germany) with hydrogen peroxide and used for protein isolation described below.

**Western blot**

Whole cell protein lysates were prepared using RIPA buffer. Nuclear protein lysates were extracted using the NE-PER Nuclear and Cytoplasmic Extraction Reagents (Thermo Fisher Scientific) supplemented with the Complete Protease Inhibitor Cocktail (Roche, Basel, Switzerland) according to the manufacturer´s instructions. Protein concentration was determined by Lowry method using DC™ Protein Assay (Bio-Rad, CA, USA). Proteins were separated by electrophoresis on Bolt™ 4-12% Bis-Tris Plus Gels (Thermo Fisher Scientific) and transferred to a nitrocellulose membrane (Bio-Rad). Non-specific binding was blocked with PBS containing 5% dry milk for 1 hour. The membrane was probed overnight with a primary antibody. Membrane-bound primary antibodies were detected using appropriate secondary antibodies conjugated with horseradish peroxidase. Membrane-bound antibody complexes were visualized using Clarity™ ECL Western Blotting Substrate Kit (Bio-Rad), SuperSignal™ West Pico Chemiluminescent Substrate Kit and/or SuperSignal™ West Femto Maximum Sensitivity Substrate Kit (Thermo Fisher Scientific) followed by exposition to X-ray films or to Uvitec Mini HD6 documentation system (Uvitec, UK). All primary and secondary antibodies are listed below:

| **Primary and secondary antibodies** | | |
| --- | --- | --- |
| Antibody | Producer (Cat. No.) | Dilution |
| Jak2 (D2E12) XP® Rabbit mAb^MC^ | Cell Signaling Technology (3230) | 1:1000 |
| Phospho-Jak2 (Tyr1007/1008) antibody^MC^ | Cell Signaling Technology (3771) | 1:1000 |
| STAT1 antibody^PC^ | Cell Signaling Technology (9172) | 1:1000 |
| Phospho-Stat3 (Tyr705) (D3A7) XP® Rabbit mAb^MC^ | Cell Signaling Technology (9145) | 1:2000 |
| Anti-Stat3 Antibody (F-2)^MC^ | Santa Cruz (sc-8019) | 1:200 |
| STAT5 (C-17) ^PC^ | Santa Cruz (sc-835) | 1:200 |
| Purified Mouse Anti-Stat1 (pY701) ^MC^ | BD Transduction Laboratories (612132) | 1:1000 |
| Purified Mouse Anti-Human Stat5 (pY694) ^MC^ | BD Transduction Laboratories (611964) | 1:1000 |
| Anti-TATA binding protein TBP antibody^PC^ | Abcam (ab63766) | 1:1000 |
| Monoclonal Anti-GAPDH antibody produced in mouse^MC^ | Sigma-Aldrich (G8795) | 1:10000 |
| Monoclonal Anti-β-Actin antibodyproduced in mouse^MC^ | Sigma-Aldrich (A5316) | 1:4000 |
| Goat Anti-Mouse IgG (H + L)-HRP | Bio-Rad (1706516) | 1:3000 |
| Goat Anti-Rabbit IgG (H+L)-HRP | Bio-Rad (1706515) | 1:4000 |

**Cell metabolism**

Oxygen consumption rate (OCR) and extracellular acidification rate (ECAR) under baseline and stressed conditions were determined using the Seahorse XFp Cell Energy Phenotype Test Kit on the Seahorse XFp Analyzer (Agilent Technologies, Inc., CA, USA). All tests were performed according to manufacturer’s instructions. Cells were plated in XF RPMI medium pH 7.4, at a density of 40,000 cells/well (Ba/F3) or 50,000 (c-kit BMCs) in XFp tissue culture plates coated with CellTak (Corning GmbH, Wiesbade, GER) according to the Agilent Seahorse protocol for the seeding of suspension cells. All of the Seahorse measurements were done at least in biological triplicates and three technical replicates.

**ATP and NAD+/NADH measurement**

The quantitative bioluminescent determination of Adenosine 5’-triphosphate (ATP) was determined by the ATP Bioluminescent Assay Kit (Sigma-Aldrich, MO, USA) according to manufacturer’s instructions. The ratio of total oxidized and reduced nicotinamide adenine dinucleotides (NAD+/ NADH) was measured by the NAD/NADH-Glo Assay (Promega, WI, USA) according to manufacturer’s instructions. All measurements were done at least in biological triplicates and three technical replicates.

**2DG cultivation assay**

The 2-deoxy-D-glucose (2DG) experiment, 30.000 Ba/F3 cells were seeded per well into a 24 well plate (in triplicate). Cells were counted, passaged and treated every second or third day with PBS (controls) or 1mM 2DG. All measurements were done at least in biological triplicates and three technical replicates.

**Stable isotope tracing**

Ba/F3 cells - EV, NPAT-JAK2, with and w/o IL-3 and PAX5-JAK2, with and w/o IL-3 (3.00E+05 cells/mL and PJ -IL-3: 6.00E+05 cells/mL) were cultured in RPMI 1640 media, 10% FBS, 1% antibiotics, supplemented with 11mM 13 C_5_-glucose (Cambridge Isotope Laboratories, Massachusetts, USA) for 24 hours. Cells were collected and metabolites from cells were immediately extracted using an already established procedure^1^. Cells washed with ice-cold 0.9% saline solution and the metabolites were extracted in 1:1:1 pre-chilled methanol, HPLC-grade water containing 1 mg/mL D6-glutaric acid (C/D/N isotopes) and chloroform. After shaking and centrifuging, the upper aqueous phase was collected and evaporated in vacuum. Details on polar metabolite derivatization, data acquisition, and gas chromatography-mass spectrometry (GS/MS) analysis can be found in Kluvkova K., et al^2^.

**Statistical analysis**

GraphPad Prism software was used for statistical calculations (GraphPad Software, San Diego, CA, USA).

**Supplementary Information**

**NPAT-JAK2 fusion gene description**

The NPAT-JAK2 fusion was identified by whole transcriptome sequencing and verified by Sanger sequencing both at the genomic and mRNA levels (Supplementary Figure 1A).

At the genomic level intron 10 of the NPAT gene was fused to incomplete exon 18 of the JAK2 gene and 27 non-templated nucleotides (NN) were inserted at the junction. At the mRNA level the partial NPAT intron 10 was spliced out utilizing a neo-acceptor-splice site formed by its last two nucleotides involved in the fusion (AG). Thus, the 27 NN were retained in the mRNA between the complete NPAT exon 10 and partial JAK2 exon 18, while the reading frames of both partner genes remained in frame (NPAT reference NM_002519, JAK2 reference NM_004972).

The predicted encoded protein involves 302 N-terminal amino acids of NPAT and 343 C-terminal amino acids of JAK2 protein including complete JAK2-kinase domain (Supplementary Figure 1B).

Supplementary references

1. Hollinshead KER, Munford H, Eales KL, et al. Oncogenic IDH1 Mutations Promote Enhanced Proline Synthesis through PYCR1 to Support the Maintenance of Mitochondrial Redox Homeostasis. *Cell Rep.* 2018;

2. Kľučková K, Thakker A, Vettore L, et al. Succinate dehydrogenase deficiency in a chromaffin cell model retains metabolic fitness through the maintenance of mitochondrial nadh oxidoreductase function. *FASEB J.* 2020;34(1):.

**Supplementary Figures:**

**Supplementary Figure 1. NPAT-JAK2 structure**

(A) Sequences of the junction region within the NPAT-JAK2 fusion gene (gDNA) and transcript (cDNA). Violet triangles separate codons within the chimeric reading frame. Inserted non-templated nucleotides (NN) are highlighted by dotted-line box.

(B) A schema of the NPAT-JAK2 protein. Structure of the NPAT protein is depicted according to Sagara et al., J Biochem 2002. Nine amino acids at the fusion point are encoded by 27 NN.

NCR - near central region; NLS1-3 - nuclear localization signals 1-3; FERM - four-point-one, ezrin, radixin, moesin domain; SH2 - Src homology domain; PK – pseudokinase domain; K – kinase domain. (C) Schematic comparison of JAK2 fusion partner in NPAT-JAK2 and PAX5-JAK2


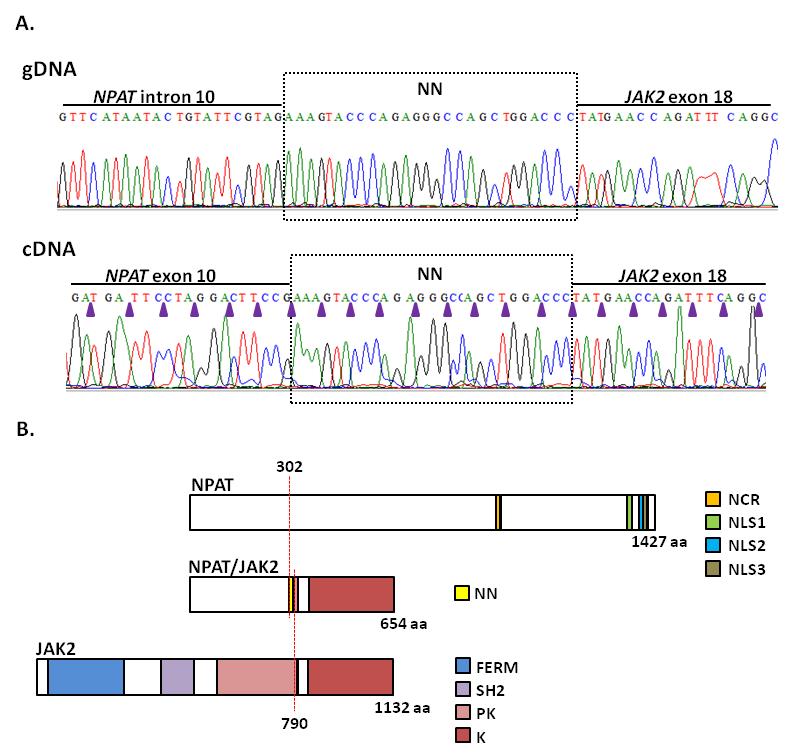


C.


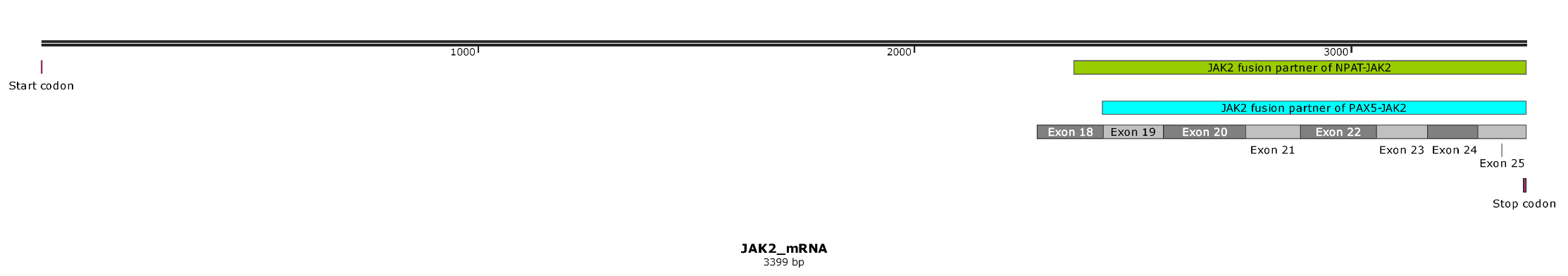
**Supplementary Figure 2. Detection of changes in cellular signaling upon NPAT-JAK2 expression**

Due to initial higher level of STAT5 phosphorylation already in parental Ba/F3 cells, we used another cell model to confirm the downstream regulation of STAT5 by NPAT-JAK2.

B-precursor leukemic cell line REH was transduced with pWCC19 (with (NJ, NJ^K882E^ or without an insert (EV))) following the sorting of GFP-positive cells. Protein extraction for western blot was performed prior the detection of NPAT-JAK2 fusion protein and STATs proteins. As a control, NPAT-JAK2-transformed Ba/F3 cells were used.







P-STAT5

STAT5

80kDa

80kDa

EV

NJ

NJ

**K882E**

NJ

GAPDH

NPAT-JAK2

70kDa

REH

Ba/F3








**Supplementary Figure 3. Statistical analysis of protein detection by western blot**

This supplementary figure represents normalized expression values of all proteins detected in Fig. 1

C i and C ii. All proteins were detected in three independent biological experiments by western blot. Densitometry was provided by Uvitec Mini HD6 documentation system and normalized to housekeeping protein. Statistical analysis was done by ANOVA. Asterisks represent the p-values: * ≤0.05; **≤0.01 and ***≤0.001.
